# Schizophrenia and schizoaffective disorder: Length of stay and associated factors

**DOI:** 10.4102/sajpsychiatry.v30i0.2237

**Published:** 2024-04-22

**Authors:** Ladawa Y. Goga, Belinda S. Marais

**Affiliations:** 1 Department of Psychiatry, Faculty of Health Sciences, University of the Witwatersrand, Johannesburg, South Africa

**Keywords:** schizophrenia, schizoaffective disorder, length of inpatient stay, readmission, substance use, placement, deinstitutionalisation

## Abstract

**Background:**

Patients with schizophrenia and schizoaffective disorder often require longer admissions.

**Aim:**

To explore length of stay (LOS) and associated factors of patients with schizophrenia and schizoaffective disorder, admitted to a public sector specialised psychiatric hospital, over a 4-year period.

**Setting:**

The study was conducted at Tara Hospital in Johannesburg.

**Methods:**

A retrospective record review of 367 adult schizophrenia and schizoaffective disorder patients admitted between 01 January 2015 and 31 December 2018. Average LOS was calculated and the proportion of short-stay (< 30 days), medium-stay (31–90 days) and long-stay (> 90 days) admissions determined. Sociodemographic, clinical and admission outcome data were collected and analysed from a randomly selected subset of patients in each LOS category.

**Results:**

Mean LOS was 128 days (median 87, interquartile range [IQR] 49–164, range 0–755 days). A significantly greater proportion had long-stay admissions (*p* < 0.001). Male gender (*p* = 0.018), being unmarried (*p* = 0.006), treatment resistant (*p* < 0.001) and on clozapine (*p* = 0.009) were factors found to have a significant association with long-stay admissions. Rates of unemployment (> 80%), comorbid substance use disorders (> 40%), medical illnesses (> 40%), antipsychotic polypharmacy (> 40%) and readmissions (> 80%) were high. Most (> 80%) were discharged.

**Conclusion:**

Long-stay admissions were frequently required for patients with schizophrenia and schizoaffective disorder admitted to Tara Hospital.

**Contribution:**

This study highlights factors associated with long-stay admissions in patients with schizophrenia and schizoaffective disorder. More research is needed into whether increased access to community-based services, such as residential and daycare facilities, outpatient substance rehabilitation programmes and dual diagnosis clinics, could translate into shorter admissions, less frequent relapses and improved outcomes in this population.

## Introduction

Schizophrenia spectrum disorders are serious mental illnesses (SMIs), often associated with significant morbidity and functional impairment.^1^ The economic burden of schizophrenia in particular has been found to be significant, with inpatient care comprising most of the direct healthcare costs.^2,3,4^ Schizophrenia accounts for the majority of inpatient psychiatric admissions and has been found to be the most common diagnosis when investigating the profile of long-stay psychiatric patients.^5,6,7^ Schizoaffective disorder shares similar clinical characteristics to schizophrenia, but with an affective component. There has been a longstanding debate regarding the classification of schizoaffective disorder and its diagnostic validity and reliability, and as such less studies exist regarding schizoaffective disorder.^8,9,10^ It has been further noted in the literature that many of those diagnosed with schizoaffective disorder later receive a diagnosis of schizophrenia.^8^ Nevertheless, schizoaffective disorder has also been associated with longer hospitalisations.^11^ Other factors found to be associated with increased length of stay (LOS) are male gender, ethnicity, unemployment, and accommodation status or homelessness.^5,12^ Illness severity in patients with schizophrenia and schizoaffective disorder has been linked to more frequent relapses and to longer hospital admissions.^11,13^ Approximately 40% of patients with schizophrenia do not achieve complete remission and have residual positive symptoms.^14^

Treatment resistance, in particular persistent psychotic symptoms, as well as treatment with more than one antipsychotic, have been found to be associated with prolonged LOS.^15^ Higher antipsychotic doses and more complex drug treatments have also been associated with longer LOS.^11^ Substance use has been shown to play a role in LOS, as well as the frequency of relapses and readmissions. High-frequency mental care users have been found to be more likely to admit to lifetime substance use, and cannabis use in particular has been found to predict increased number of subsequent admissions and lead to longer admissions.^16,17,18^ There are, however, conflicting results in the literature, in that some studies have found that patients with chronic psychotic disorders and comorbid substance use disorders have shorter LOS as compared to the nonsubstance users, although a greater number of hospitalisations and total hospital days.^19,20^ Active medical comorbidity is another factor found to be associated with increased LOS in patients with schizophrenia.^21^ Regarding the trend in LOS, this differs between developing and developed countries. Though there seems to be a gradual trend of increasing LOS of patients with mental illnesses in developing countries, the LOS for those with SMIs in developed countries has reduced significantly in the last three decades, and this has been attributed to improved community-based care.^6,22,23^

Deinstitutionalisation refers to a change in psychiatric care setting from long-term treatment in specialised facilities back to treatment within the community.^24^ While deinstitutionalisation may reduce costs by reducing hospital stay, other costs, such as that of developing efficient community-based services, and consequences must be considered.^25,26^ Failure to couple deinstitutionalisation with improved community care has been shown to lead to further problems, namely increased relapses and readmissions, incarcerations, homelessness, increased suicide and mortality rates.^25,27^ In the South African context, attempts at deinstitutionalisation occurred but without adequate development of community psychiatric services and psychosocial rehabilitation facilities.^28,29^ This discrepancy was noted in the National Mental Health Policy Framework and Strategic Plan 2013–2020, and the Life Esidimeni tragedy of 2016 further demonstrated the consequences of a failed and poorly planned attempt at deinstitutionalisation.^26,29,30^ Moreover, mental health budget allocation deficiencies generally, as well as the disparity between funding of hospital psychiatric services versus community mental healthcare services have been highlighted as issues needing to be addressed and rectified.^26,28,29^ Length of stay is an important marker for healthcare providers and hospital administrators and must be considered for financial planning, as it impacts on direct hospital costs.^5,31^ Interventions that may reduce LOS include placement, step-down facilities and transitional care facilities.^32^ Community-based rehabilitation facilities, based at primary care level, have been shown to improve functional outcomes in patients with schizophrenia. Assertive community-based treatment (ACT) and modified assertive interventions initiated in the Western Cape in response to pressure for inpatient beds and crisis (premature) discharge policies, which serve as community-based interventions to reduce LOS and readmissions, have proven to be successful.^33^ While community psychiatric services in our setting are lacking, there also exists a shortage of specialised psychiatric hospitals, with a reduction of bed numbers over the years. It is thus a necessity to investigate the trends in LOS of patients with schizophrenia and schizoaffective disorder in South African psychiatric hospitals for effective financial planning and structuring of health systems to occur.

## Aim and objectives

This study aimed to explore LOS and associated factors of adults diagnosed with schizophrenia and schizoaffective disorder, admitted to Tara H. Moross Hospital, a specialised psychiatric hospital, over a 4-year period.

The study objectives are listed as follows:

1. To quantitate length of hospital stay over the study period from 2015 to 2018, and determine the proportion of short-stay (0–30 days), medium-stay (31–90 days) and long-stay (> 90 days) admissions.
2. To determine the trend with regard to LOS over the study period.
3. In a subgroup of patients, to explore associated sociodemographic factors, clinical factors and admission outcomes, and determine whether any relationship exists between these factors and the three categories of LOS.

## Research methods and design

### Study design and setting

This was a retrospective record review conducted at Tara Hospital, one of the only three public sector specialised psychiatric hospitals in Gauteng. Tara Hospital receives referrals from various surrounding acute hospitals in Johannesburg and provides inpatient and outpatient services. It has both specialised wards and biological wards, the latter being for treatment of adult patients with a primary diagnosis of an SMI requiring medium-term hospitalisation. There are three biological wards, consisting of 80 beds. Referrals are placed on a waiting list for admission, with waiting times being influenced by the number of referrals, bed pressures at the acute units, as well as inpatient turnaround times, that is, LOS. Tara Hospital has no formalised policy for crisis (premature) discharges. No assertive community interventions are currently available for patients discharged from public sector hospitals in and around Johannesburg.

### Participants

Adult patients diagnosed with schizophrenia or schizoaffective disorder and admitted to any of the biological wards at Tara Hospital during the 4-year study period of 01 January 2015 to 31 December 2018 were included in the study.

Patients who were less than 18 years, still admitted to Tara Hospital at the time of data collection or where the primary diagnosis was unclear, were excluded.

### Data collection

Data regarding LOS (objectives 1 and 2) were obtained from the admission registers (which record date of admission and discharge, and diagnosis) in each of the biological wards. Patients’ clinical files (with discharge summaries and clinical notes) were retrieved from the hospital’s registry department, to collect data regarding the patient’s sociodemographic and clinical factors (objective 3).

### Statistical analysis

Data were analysed descriptively using charts and tables. Statistical analyses were conducted in R software (version 3.5.1; www.R-project.org). The normal distribution of the data was checked using the Shapiro–Wilk test and examining Q-Q plots. All data were categorical, and nonparametric analyses were conducted. Tests were two-tailed and model significance was set at 0.05.

Objective 1: The mean LOS, standard deviation (s.d.), range, median and interquartile range (IQR) were calculated. Furthermore, the percentage of patients in the total study population that had short-, medium- and long-stay admissions was reported.

Objective 2: Median and IQR were calculated for every 6 months over the 4-year study period to determine the trend. Additionally, the proportion of patients that had short-, medium- and long-stay admissions was calculated in each 6-month period and analysed using chi-squared contingency table analyses to assess whether the proportions of admissions deviated from chance.

Objective 3: Regarding the relationship between sociodemographic and clinical factors and LOS, simple random sampling was used to select an equal number of patients from each of the three LOS categories.

Statistical significance was calculated and was expected with a minimum sample of 75 patients. Thus, data were collected from a minimum of 25 patient files from each category (short, medium and long stay). All data were categorical, and a Pearson chi-squared (χ^2^) analysis was used to determine whether the distribution of patients in the three LOS groups deviated from chance.

### Ethical considerations

Approval was obtained from the University of the Witwatersrand (WITS) Human Research Ethics Committee (HREC), clearance no. M191192, and Tara Hospital’s Research Committee and Chief Executive Officer. All participants were allocated a study number and data were anonymised, thereby ensuring privacy and confidentiality of all patients’ personal information.

## Results

A total of 367 patients met the inclusion criteria and were included in the study.

### Objective 1

**FIGURE 1:**
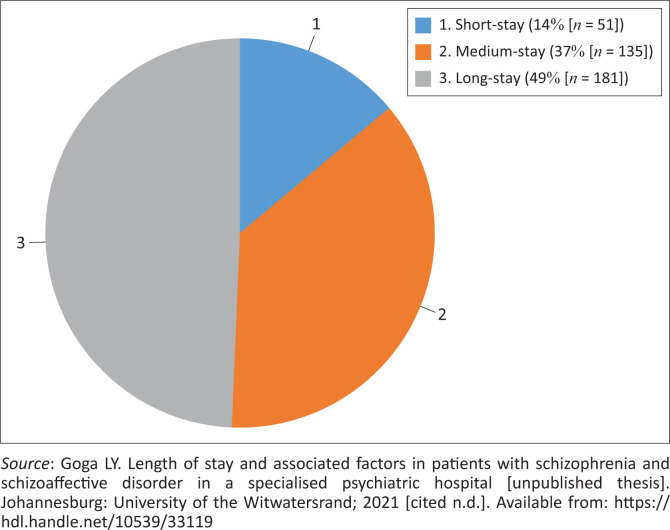
Proportion of short-, medium- and long-stay admissions of schizophrenia and schizoaffective disorder patients admitted to Tara Hospital from 2015 to 2018.

The mean LOS was 128.24 days (s.d. = 120.89; range 0–755). Median LOS was 87 days (IQR 45–164.5 days). The proportion of short-, medium- and long-stay admissions is shown in Figure 1.^34^ A significantly greater percentage of patients had long-stay admissions, followed by medium-stay and then short-stay admissions ($\chi_{2}^{2}$ = 71.04, *p* < 0.001).

### Objective 2

**FIGURE 2:**
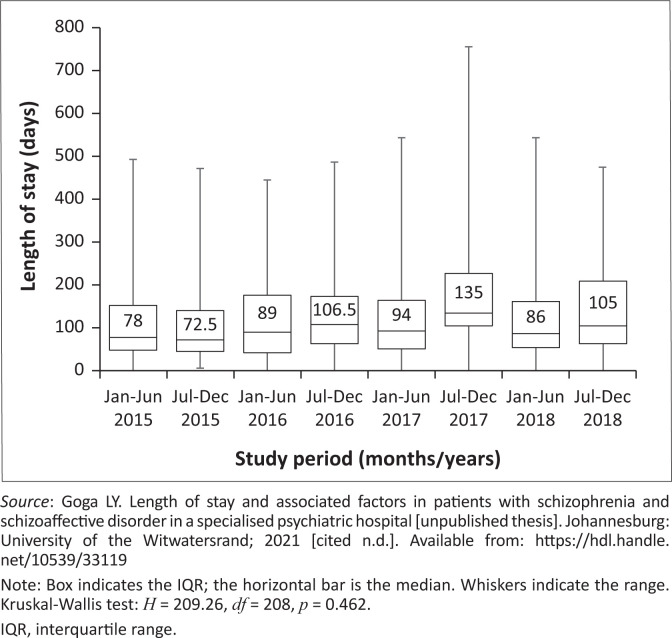
Length of stay trend of schizophrenia and schizoaffective disorder patients admitted to Tara Hospital every 6 months from 2015 to 2018.

**FIGURE 3:**
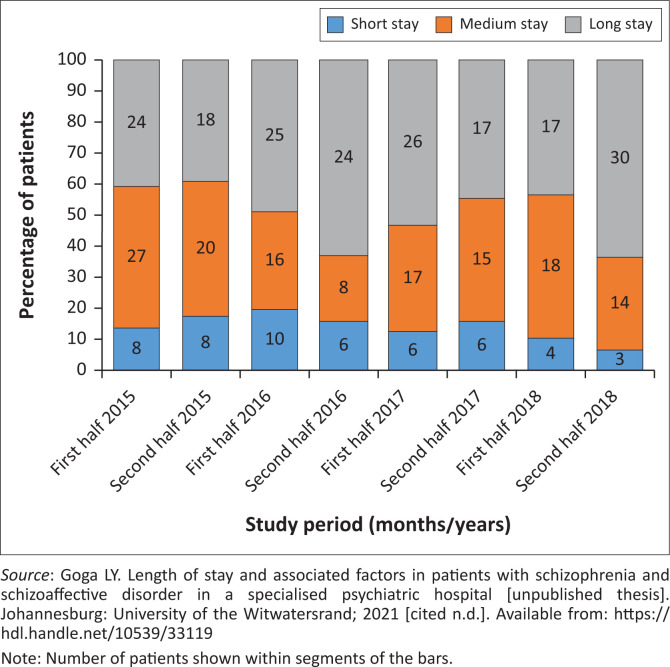
Short-, medium- and long-stay admissions of schizophrenia and schizoaffective disorder patients at Tara Hospital every 6 months from 2015 to 2018.

The trend with regards to LOS from 2015 to 2018 is shown in Figure 2.^34^ The shortest median stays were in 2015 and the longest median stay was in the second half of 2017. The range was also greatest in 2017. Figure 3^34^ indicates the percentage of patients that had short-, medium- and long-stay admissions over the course of the study period. A significantly greater percentage had long-stay admissions, and the least had short-stay admissions:

$$\chi_{14}^{2}=91.64,p<0.001$$ [Eqn 1]

### Objective 3

Sociodemographic, clinical and admission outcome data were available for 25 short-stay, 26 medium-stay and 25 long-stay patients and are shown in Table 1,^34^ Table 2^34^ and Table 3.^34^

**TABLE 1:** Sociodemographic factors of short-, medium- and long-stay schizophrenia and schizoaffective disorder patients admitted to Tara Hospital from 2015 to 2018.

Sociodemographic factors	Short stay (*N* = 25)		Medium stay (*N* = 26)		Long stay (*N* = 25)		χ^2^ value	*p*
	*n*	%	*n*	%	*n*	%		
**Age (years)**	-	-	-	-	-	-	$\chi_{4}^{2}$ = 6.08	0.193
18–35	10	40	10	38.5	16	64	-	-
36–50	9	36	10	38.5	8	32	-	-
> 50	6	24	6	23	1	4	-	-
**Gender**	-	-	-	-	-	-	$\chi_{2}^{2}$ = 8.05	**0.018**
Male	8	32	14	53.8	18	72		
Female	17	68	12	46.2	7	28		
**Ethnicity**	-	-	-	-	-	-	$\chi_{6}^{2}$ = 3.58	0.734
African people	16	64	21	80.8	20	80	-	-
Caucasian people	7	28	4	15.4	3	12	-	-
Mixed race people	1	4	1	3.8	1	4	-	-
Indian people	1	4	0	0	1	4	-	-
**Nationality**	-	-	-	-	-	-	$\chi_{6}^{2}$ = 2.08	0.353
South African	22	88	25	96.2	21	84	-	-
Non-South African	3	12	1	3.8	4	16	-	-
**Marital status**	-	-	-	-	-	-	$\chi_{4}^{2}$ = 14.50	**0.006**
Married	8	32	2	7.7	0	0	-	-
Unmarried	16	64	22	84.6	25	100	-	-
Unknown	1	4	2	7.7	0	0	-	-
**Referral source**	-	-	-	-	-	-	$\chi_{2}^{2}$ = 1.01	0.604
Public hospital	24	96	25	96.2	25	100	-	-
Private	1	4	1	3.8	0	0	-	-
**Highest level of education**	-	-	-	-	-	-	$\chi_{8}^{2}$ = 9.17	0.328
No formal schooling	1	4	2	7.7	0	0	-	-
Primary	6	24	11	42.3	5	20	-	-
Secondary	11	44	10	38.5	16	64	-	-
Tertiary	4	16	1	3.8	3	12	-	-
Unknown	3	12	2	7.7	1	4	-	-
**Employment status**	-	-	-	-	-	-	$\chi_{4}^{2}$ = 2.48	0.648
Employed	2	8	2	7.7	3	12	-	-
Unemployed	23	92	24	92.3	21	84	-	-
Unknown	0	0	0	0	1	4	-	-

*Source:* Goga LY. Length of stay and associated factors in patients with schizophrenia and schizoaffective disorder in a specialised psychiatric hospital [unpublished thesis]. Johannesburg: University of the Witwatersrand; 2021 [cited n.d.]. Available from: https://hdl.handle.net/10539/33119

**TABLE 2:** Clinical factors of short-, medium- and long-stay schizophrenia and schizoaffective disorder patients admitted to Tara Hospital from 2015 to 2018.

Clinical factors:	Short-stay (*N* = 25)		Medium-stay (*N* = 26)		Long-stay (*N* = 25)		χ_2_ value	*p*-value
	*n*	%	*n*	%	*n*	%		
**Comorbidity:**								
Substance (including alcohol) use disorder	-		-	-	-	-	$\chi_{2}^{2}$ = 0.49	0.784
Yes	12	48	11	42.3	13	52	-	-
No	13	52	15	57.7	12	48	-	-
Other comorbid psychiatric disorder	-	-	-	-	-	-	$\chi_{2}^{2}$ = 7.86	0.020
Yes	8†	32	11‡	42.3	2§	8	-	-
No	17	68	15	57.7	23	92	-	-
Comorbid medical disorder							$\chi_{2}^{2}$ = 1.31	0.5200
Yes	10¶	40	13††	50	14‡‡	56	-	-
No	15	60	13	50	11	44	-	-
**Illness severity factors:**								
Number of antipsychotic medications on discharge	-	-	-	-	-	-	$\chi_{2}^{2}$ = 2.47	0.292
One	13	52	13	50	8	32	-	-
Two or more	12	48	13	50	17	68	-	-
Antipsychotic treatment resistance - ie. failed > 2 antipsychotic trials and/or on clozapine	-	-	-	-	-	-	$\chi_{2}^{2}$ = 22.09	**< 0.001**
Yes	4	16	9	34.6	20	80	-	-
No	21	84	17	65.4	5	20	-	-
Discharged on Clozapine§§	-	-	-	-	-	-	$\chi_{2}^{2}$ = 9,33	0.009
Yes	2	8	4	15.4	12	48	-	-
Clozapine resistance - ie. of those on clozapine (*), documented residual psychosis on discharge	-	-	-	-	-	-	$\chi_{2}^{2}$ = 3.00	0.223
Yes	2	100	1	25	6	50	-	-
No	0	0	3	75	6	50	-	-
Previous psychiatric admissions - ie. readmission vs first / index admission	-	-	-	-	-	-	$\chi_{2}^{2}$ = 2.34	0.310
Yes	21	84	21	80.8	24	96	-	-
No	4	16	5	19.2	1	4	-	-
Previously placement	-	-	-	-	-	-	$\chi_{2}^{2}$ = 3.77	0.152
Yes	0	0	1	3.8	3	12	-	-
No / Unknown	25	100	25	96.2	22	88	-	-

† , 3x personality disorders; 2x neurocognitive disorders; 2x trauma/stressor related; 1x anxiety;

‡ , 2 x personality disorders; 1 x mood disorder; 4 x neurocognitive disorder; 4 x other;

§ , 2x personality disorders;

¶ , 1 x epilepsy; 3 x HIV; 1 x previous head injury; 5 x other medical disorder;

†† , 1 x diabetes mellitus; 4 x HIV; 8 x hypertension;

‡‡ , 4 x diabetes mellitus; 7 x hypertension; 2 x previous head injury; 1 x other medical disorder;

§§ , Figures in italics = % of those on clozapine, not of total study population.

**TABLE 3:** Admission outcomes of short-, medium- and long-stay schizophrenia and schizoaffective disorder patients admitted to Tara Hospital from 2015 to 2018.

Admission outcomes	Short stay (*N* = 25)		Medium stay (*N* = 26)		Long stay (*N* = 25)		χ^2^ value	*p*-value
	*n*	%	*n*	%	*n*	%		
Discharged home	20	80	24	92.3	20	80	$\chi_{2}^{2}$ = 1.95	0.337
Placement was however recommended during admission	5	*25*	8	*33*	9	*45*	$\chi_{2}^{2}$ = 1.75	0.416
Discharged to placement	2	8	2	7.7	4	16	$\chi_{2}^{2}$ = 0.84	0.656
Other, *i.e. transferred to another facility (Sterkfontein Hospital), or discharged in absentia (absconded)*	3	12	0	0	1	4	$\chi_{2}^{2}$ = 3.80	0.150

*Source:* Goga LY. Length of stay and associated factors in patients with schizophrenia and schizoaffective disorder in a specialised psychiatric hospital [unpublished thesis]. Johannesburg: University of the Witwatersrand; 2021 [cited n.d.]. Available from: https://hdl.handle.net/10539/33119

Figures in italics represent the percent (%) of those discharged home, not total study population.

## Discussion

### Objective 1

The LOS in this study was increased compared to a previous study done at Tara Hospital, where the median LOS for schizophrenia patients in 2009 was 53 days (IQR 30–94).^5^ Increased psychiatric patient numbers in Gauteng have been reported over the years between these two studies, which may have resulted in Tara biological ward referrals being reserved for more severely ill and treatment-resistant patients compared to previous years, because of increased pressure for beds and more rapid patient turnaround times in the acute hospitals.^29^ In another local study which reviewed the profile of patients, the majority of whom were diagnosed with a primary psychotic disorder, who absconded from Sterkfontein Hospital in 2008, the mean LOS of (nonforensic) patients was 100 days. However, 29% were not returned after absconding and therefore discharged in absentia, rather than because of being well and ready for discharge.^35^ A Canadian study conducted between 2005 and 2015 found a mean LOS of 96 days for inpatients with schizophrenia.^17^ Target LOS for medium- to long-stay psychiatric hospitals in South Africa is 180 days according to the Department of Health Norms Manual for Severe Psychiatric Conditions and as calculated by the World Health Organization (WHO).^36^ The proportion of patients in this study with long-stay admissions was significantly higher (*p* < 0.001) than the other categories of LOS, which was not an unexpected finding considering their clinical profile and that patients are generally referred to Tara Hospital (as opposed to being discharged from the acute units) when it is anticipated that they will need longer admissions.

### Objective 2

Regarding the trend in LOS, the proportion of long-stay admissions was significantly greater (*p* < 0.001) in all 4 years of the study. Additionally, LOS figures were lowest in 2015 and higher in subsequent years, though this was not statistically significant. Generally, patients may be suitable to go to placement facilities, where they would receive further psychosocial and functional rehabilitation, sooner than they may be considered ready to be discharged home. Thus the closure of the various Life Esidimeni facilities (2016), which Tara Hospital relies on for placement transfers, may have been a contributing factor.^30^ In a previous national situation analysis, according to existing service indicators, the bed to population ratio for medium- to long-term psychiatric facilities was 35:100 000, including Life Esidimeni beds, as opposed to 16:100 000 if these beds were excluded.^37^ In other words, following the closure of Life Esidimeni, there was a significant reduction in available long-term psychiatric beds, placing an additional burden on a medium-term facility such as Tara Hospital.

### Objective 3

In terms of sociodemographics, gender and marital status differed significantly between the three categories of LOS, with the long-stay group having the most male and unmarried patients. This is in keeping with a British study which examined LOS of psychiatric inpatients and found a positive association between male gender and increased LOS, but not between marital status and LOS.^12^ Marital status is a good prognostic factor and associated with increased social support, with married and/or cohabitating schizophrenia spectrum disorder patients having been found to have better quality of life and be less likely to be living alone than single, separated, divorced and/or widowed patients.^38^ Therefore, it may be in this study that married patients were more likely to have shorter admissions because of better support and conversely with the unmarried patients. Age, ethnicity, nationality, level of education, employment and referring hospital did not differ significantly between the three categories of LOS in this study. The British study, however, found a positive association between unemployment and African ethnicity and increased LOS.^12^ The lack of a significant association regarding employment status may be explained by the fact that majority of the patients included in this study were unemployed, which is partly a reflection of the unemployment rates in South Africa, as well as perhaps the severity of their illness. The majority of patients in all categories of LOS were also readmissions. Therefore, high rates of unemployment in all patients may have been expected. There were high rates (over 40%) of comorbid substance use disorders, in all LOS groups, in keeping with literature which has shown that substance use is common in people with schizophrenia and other psychotic disorders.^39,40,41^ However, substance use disorders did not predict a longer LOS in this study. Other psychiatric comorbidities also occur frequently in those with schizophrenia, and this comorbidity is often associated with a more severe illness and poorer outcomes.^41,42^ This study’s finding of significantly higher rates of other psychiatric comorbidities in the short- and medium-stay groups as compared to the long-stay group was therefore unexpected. A possible explanation is that in the long-stay patients, because of greater illness severity and treatment resistance, comorbid psychiatric diagnoses may not have been as easily detected as with the other patients. Rates of personality disorders were similar though, among the three groups, consistent with a recent review article which did not find convincing evidence that comorbid personality disorders worsened the course of illness in schizophrenia as opposed to that of other SMIs.^43^ Medical comorbidities were common and occurred similarly in all three categories of LOS, with hypertension being most common, followed by human immunodeficiency virus (HIV) and diabetes mellitus. In the long-stay group, hypertension was often comorbid with diabetes. In these patients, this could possibly indicate metabolic syndrome, which can also be associated with dyslipidaemia, being overweight and an increased risk of heart disease. This group also had the highest rates of treatment resistance, and being on clozapine. Metabolic syndrome and other cardiovascular risk factors are highly prevalent in patients with schizophrenia, contributing to an increased risk for premature mortality. In addition to unhealthy diet, sedentary lifestyle and smoking, atypical antipsychotics can also have a negative impact on cardiometabolic risk factors.^44^ Regarding HIV, a large Danish study, of 2.6 million participants over 17 years, concluded that a diagnosis of HIV was associated with a significantly increased risk of developing schizophrenia or an episode of psychosis.^45^ The impact of comorbid SMI and HIV, according to a recent review, is unclear, with limited evidence that it is associated with worse clinical outcomes.^46^ In this study, HIV was the second most common medical comorbidity with similar distribution in the short- and medium-stay groups, but none in the long-stay group. A possible explanation for this may be that the long-stay patients were less sexually active, as a result of having a more severe illness, with more severe negative symptoms and impairments in social functioning, compared to the other patients. Regarding severity variables, the long-stay group had the highest percentage of patients on antipsychotic polypharmacy, but the difference was not statistically significant. The rate of antipsychotic polypharmacy was in fact found to be high in all three groups. This is in keeping with a recent study in India which found a high rate (44%) of antipsychotic polypharmacy, with the most common reason being use of a depot with an oral antipsychotic.^47^ The frequency of depot antipsychotic use was not, however, captured in the current study. Treatment resistance rates were significantly higher in the long-stay group as compared to the other categories of LOS. However, only about half of the patients with treatment resistance, in all three groups, were discharged on clozapine. This underutilisation of clozapine is in keeping with that described in the literature.^48^ Nonetheless, the proportion of patients discharged on clozapine was still significantly higher in the long-stay group compared to the other two groups. The difference between groups with regards to clozapine resistance, however, was not significant, and there were patients discharged with residual psychosis despite treatment with clozapine in all LOS groups. It is estimated in the literature that 40%–70% of patients with treatment-resistant schizophrenia do not respond to clozapine antipsychotic monotherapy.^49^ Most patients in all LOS groups were readmissions, with the highest rate in the long-stay group. Multiple relapses are frequent in patients with schizophrenia, and it has been suggested that psychosis may be neurotoxic and that relapses lead to disease progression and impairment of treatment response.^50^

Based on the patients’ clinical profile, it is likely that many may have benefitted from referral to residential placement facilities; however, the majority were discharged home, in all LOS groups, and not placed. Similarly, the proportion of patients who had previously been admitted to placement facilities was very low. This is indicative of the scarcity of community-based residential care beds in this setting.^37,51^ Placement was recommended in just under half of the patients who were discharged home in the long-stay group, and even in the short- and medium-stay groups, it was recommended for a quarter and a third, respectively. In these cases, patient and/or family refusal may have been the reason, but it may also have been because of long waiting lists for placement. To what extent though the shortage of beds at placement facilities, and certainly also the closure of Life Esidimeni, may have contributed to longer hospital admissions cannot be determined from this study.

### Strengths and limitations

As with retrospective studies, limitations were related to recordkeeping. Missing information, such as unrecorded diagnoses or missing dates in the admission registers resulted in exclusion of patients who may have been eligible for inclusion in the study. Accuracy of the clinical information in patient records may also have been a limitation. The data for this study covered the 4-year period of 2015–2018 and thus do not describe the current LOS and associated factors of patients with schizophrenia and schizoaffective disorder in this setting. Strengths of this study were that it has provided findings in an area where research has been limited.

### Implications and recommendations

Ongoing efforts should be made to improve the management of patients with schizophrenia and schizoaffective disorder, including addressing substance use, preventing relapses and increased use of clozapine in treatment-resistant patients. Improved community-based services are essential, as well as the need for more published data on step-down facilities in South Africa.^30,52^ Such improvements may also provide cost-effective alternatives to admission in psychiatric hospitals, without negatively impacting on patient outcomes.^53,54^ The WHO Mental Health Action Plan also emphasises the need to provide comprehensive, integrated, and responsive mental health and social care services in community-based settings and to strengthen research for mental health.^55^

## Conclusion

The majority of patients with schizophrenia and schizoaffective disorder had long-stay admissions. Male gender, being unmarried, treatment-resistant and on clozapine occurred most frequently in the long-stay group. Overall rates of unemployment, substance, other psychiatric and medical comorbidities, antipsychotic polypharmacy and readmissions were high, and clozapine remains underutilised. Most patients were discharged home, despite placement often being recommended. The shortage of psychiatric residential placement, step-down and transitional care facilities likely impacts on LOS. Further and updated research in this area is required.
